# Antimicrobial resistance of commensal *Escherichia coli* from food-producing animals in Russia

**DOI:** 10.14202/vetworld.2020.2053-2061

**Published:** 2020-10-02

**Authors:** Dmitry A. Makarov, Olga E. Ivanova, Sergey Yu. Karabanov, Maria A. Gergel, Anastasia V. Pomazkova

**Affiliations:** 1 Department of Pharmaceutical Drugs for Animals, Food and Feed Safety, Russian State Center for Animal Feed and Drug Standardization and Quality, Zvenigorodskoe Highway, Russia; 2 Department of Biotechnology, Russian State Center for Animal Feed and Drug Standardization and Quality, Zvenigorodskoe Highway, Russia

**Keywords:** animals, antimicrobial resistance, commensal bacteria, critically important antimicrobials, *Escherichia coli*, multidrug resistance

## Abstract

**Background and Aim:**

Commensal *Escherichia coli* is an important indicator of antimicrobial resistance (AMR) in animals and food of animal origin. Therefore, it was recommended by the World Health Organization and OIE for inclusion in resistance surveillance programs. At the same time, the data on *E. coli* isolates from animals in Russia are scarce. The aim of this work was to determine the current prevalence of resistance and genetic markers of non-pathogenic commensal *E. coli* collected from major food-producing animals (poultry, pigs, and cows) in different regions of Russia and to compare these data with data from other countries to prioritize antimicrobials for limiting their use according to the National Action Plan

**Materials and Methods:**

Samples (n=306) were collected from biomaterial of chicken, turkey, cows, and pigs raised on 11 farms in the European part of Russia, Siberia, and North Caucasus. Isolates (n=306) of *E. coli* were tested for resistance to 11 antimicrobials from ten classes using the broth microdilution method. MICs were interpreted against EUCAST microbiological and clinical breakpoints. For data analysis and statistical processing, the AMRcloud online platform was used. The data are presented in comparison with other countries.

**Results:**

In Russia, higher levels of microbiological and clinical resistance of *E. coli* to critically important antimicrobials, including colistin, cefotaxime, and ciprofloxacin, were found compared to other countries, especially in poultry: About 30% of isolates from chicken were resistant to colistin, 8% to cefotaxime, and 88% to ciprofloxacin according to EUCAST ECOFFs. Only 10% of isolates from cows were resistant to cefotaxime. About 47% of isolates of *E. coli* from chicken had a moderate relative resistance for ampicillin and 56% for tetracycline. For most antimicrobials, isolates from cows demonstrated a lower resistance than isolates from poultry and pigs. All tested isolates from chicken, turkey, and pigs showed a simultaneous microbiological resistance to at least three classes of antimicrobials. No pan-resistant isolates were found.

**Conclusion:**

High levels of AMR of commensal *E. coli* from poultry, especially for critically important drugs, are a matter of concern and should be taken into account when choosing antimicrobials to be restricted for use in animal husbandry according to the National Action Plan.

## Introduction

Antibiotic use in animal husbandry is considered to be responsible for the emergence, selection, and spread of resistant bacteria [1]. Commensal bacteria, especially inhabitants of the intestinal tract of animals and humans, are repeatedly exposed to antibiotics and develop resistance, thus becoming an important reservoir of antibiotic resistance genes [1]. Resistant clinical pathogens pose the most immediate threat to humans, but it is becoming increasingly clear that they are not the only ones that matter. Rather, all pathogenic, commensal, and environmental bacteria, as well as mobile genetic elements and bacteriophages, form a reservoir of ARGs (resistome), from which ­pathogenic bacteria can acquire resistance through horizontal gene transfer [2]. An indirect mechanism for generating antimicrobial resistance (AMR) in pathogenic bacteria by acquiring genes from commensal bacteria may be even more efficient than direct selection [3].

Considering the above, monitoring AMR in commensal bacteria is an important part of surveillance programs. *Escherichia coli* is not only a normal part of intestinal microflora in humans and animals, but may also cause serious infections, such as gastrointestinal tract diseases, urinary tract infections, and bacteremia [4]. *E. coli* is a well-known zoonotic bacterium [5].

OIE (World Organization for Animal Health) recommends to monitor AMR of commensal *E. coli* in isolates from animals [6]. The World Health Organization (WHO) lists *E. coli* among the most common bacteria included in foodborne AMR monitoring programs [7].

The European Union (EU) includes data on commensal *E. coli* from farm animals in the annual integrated report on AMR [8,9]. *E. coli* from animals is a part of the National Antimicrobial Resistance Monitoring System (NARMS) in the USA [10]. There are a number of similar studies from other countries, including China, and Brazil [11,12].

Data on AMR of commensal *E. coli* from animals in the Russian Federation are scarce and isolates from animals are not included in AMR surveillance programs. More research in this field is needed to develop measures to minimize the spread of resistance.

The aim of this work was to determine the current prevalence of resistance and genetic markers of non-pathogenic commensal *E. coli* collected from major food-producing animals (poultry, pigs, and cows) in different regions of Russia and to compare these data with data from other countries to prioritize antimicrobials for limiting their use according to the National Action Plan [13].

## Materials and Methods

### Ethical approval

The study was approved by the Federal Service for Veterinary and Phytosanitary Surveillance (Rosselkhoznadzor).

### Sample collection

The samples (n=306) were collected in 2017-2019 from the biomaterial of farm animals that appeared to be healthy: Broiler chicken, adult turkeys, adult cows (*Bos taurus*), and feedlot pigs. The number of samples for each animal species, geographical locations of farming units, and sample types are shown in Table-1. Poultry was raised by big agricultural enterprises with several independent facilities located at a distance of several kilometers from each other. The samples were collected from each facility. Additional 32 samples of chicken, bovine, and porcine biomaterial were taken for colistin analysis using commercial Sensititre^™^ (Thermo Fischer Scientific, USA) plates (Table-2).

**Table 1:** Number of samples for each animal species, biomaterial type, and farm location.

Samples (n=306)	Poultry (n=182)		Cows (n=100) *Bos Taurus* adult	Pigs (n=24) feedlot swine

	Broiler chicken (n=163)	Adult turkey (n=19)		
Farm in Belgorod oblast №1	C-32, F-10			
Farm in Belgorod oblast №2	C-12, F-20			
Farm in Belgorod oblast №3		B-16, F-3		
Farm in Chelyabinsk oblast	C –13, F-8, E-4			F-24
Farm in Dagestan	C-20, F-5			
Farm in Yaroslavl oblast	C-14			
Farm in Novosibirsk oblast			F-15, V-10	
Farm in Penza oblast	C-23, E-2			
Farm in Tver oblast			F-15, V-10	
Farm in Voronezh oblast			V-19, F-6	
Farm in Kaluga oblast			V-19, F-6	

**Table 2:** Additional samples for colistin analysis using sensititre plates.

Samples (n=306)	Broiler chicken (n=21)	Cows (n=4) *Bos Taurus* adult	Pigs (n=7) Feedlot swine
Farm in Nenets Autonomous okrug		F – 3	
Farm in Republic of Mordovia		L-1	
Farm in Vladimir oblast	L-5		
Farm in Yaroslavl Oblast	C-5		
Farm in Moscow Oblast № 1	C-2, F-2		
Farm in Moscow Oblast №2	C-6, F-1		
Farm in Ivanovo Oblast	L-2		
Farm in Tambov Oblast	L-3		
Farm in Saint Petersburg Oblast	C-10, L-1		
Farm in Belgorod Oblast			L-6
Farm in Irkutsk Oblast			L-1
Farm in Tver Oblast			L-1

C=Carcass swab, F=Feces, E=Egg swab, B=Beak swab, V=Vagina swab, L=Liver

### *E. coli* isolation and identification

*E. coli* was isolated on Endo Agar (104044 - Merck Millipore, USA). As an enrichment medium, we used peptone broth supplemented with lactose and bile (Kessler-GRM medium, Obolensk, Russia). Incubation was carried out at 37°C for 24 h on both media. Identification of isolates was performed by the MALDI-TOF MS method using the MALDI Biotyper Microflex system (Bruker, USA), according to the Maldi Biotyper 3.0 User Manual. The identification results were verified by the API20E biochemical kit (BioMerieux, France). One isolate per each sample was obtained (n=306).

### Antimicrobial susceptibility testing (AST)

The following antimicrobial standards were purchased from Sigma-Aldrich, USA: Ampicillin, cefotaxime, chloramphenicol, ciprofloxacin, colistin, erythromycin, gentamicin, meropenem, rifampicin, streptomycin, sulfamethoxazole, and tetracycline.

AST was performed by broth microdilution according to the standard ISO [14,15] and CLSI methods [16].

For additional colistin analysis, we used a commercial Sensititre™ system (Thermo Fischer Scientific, USA) consisting of Sensititre Autoreader, Autoinoculator, Vizion, and Nephelometer modules with veterinary plate GNX3F (containing colistin). The results obtained by the manual broth microdilution method were verified using Sensititre^™^ plates.

For quality control, the following strains were used: *E. coli* ATCC 25922, *E. faecalis* ATCC 29212, and *P. aeruginosa* ATCC 27853.

## Interpretation of MICs and analysis of results

MICs were interpreted in accordance with the EUCAST Epidemiological CutOff Values (EUCAST ECOFFs) to determine microbiological resistance [17] and in accordance with the EUCAST clinical breakpoints ver. 2019 (EUCAST 2019) [18] and partly with the CLSI clinical breakpoints to determine clinical resistance [19].

To determine microbiological multidrug resistance (EUCAST ECOFFs), we used data on ten classes of antimicrobials: Penicillins (ampicillin), cephalosporins (cefotaxime), phenicols (chloramphenicol), quinolones (ciprofloxacin), polymixins (colistin), carbapenems (meropenem), aminoglycosides (streptomycin), tetracyclines (tetracycline), sulfonamides (sulfamethoxazole), and diaminopyrimidines-sulfonamides- (trimethoprim-sulfamethoxazole).

To determine clinical multidrug resistance (EUCAST 2019), we used data on eight classes: Penicillins (ampicillin), cephalosporins (cefotaxime), phenicols (chloramphenicol), quinolones (ciprofloxacin), polymixins (colistin), carbapenems (meropenem), aminoglycosides (gentamicin), and diaminopyrimidines- sulfonamides- (trimethoprim-sulfamethoxazole).

The analysis of the results, including statistical processing and visualization, was carried out using a free-access AMRcloud online platform (https://amrcloud.net). The platform was developed by the Institute of Antimicrobial Chemotherapy (Smolensk, Russia) to process user data on AMR; structural modules are modified to address the needs of researchers [20].

## Results

The prevalence of *E. coli* microbiological (EUCAST ECOFFs) and clinical (EUCAST 2019) resistance is shown in Table-3.

**Table 3:** Prevalence of antimicrobial resistance among *Escherichia coli* isolates.

Antimicrobial	Broiler chicken			Turkey		
	
	% (ECOFF)	% (EUCAST)	N of isolates	% (ECOFF)	% (EUCAST)	N of isolates
Ampicillin	49.7	49.7	163	57.9	57.9	19
Cefotaxime	8.1	7.4	149	10.5	10.5	19
Chloramphenicol	46.0	48.5	163	36.8	36.8	19
Ciprofloxacin	87.9	64.4	149	100.0	68.4	19
Colistin	33.6	33.6	149	42.1	42.1	19
Gentamicin	17.2	15.3	163	47.4	47.4	19
Meropenem	0.0	0.0	149	0.0	0.0	19
Streptomycin	59.1		149	79.0		19
Sulfamethoxazole	95.3		149	89.5		19
Tetracycline	56.4		163	89.5		19
Trimethoprim-sulfamethoxazole	85.2	4.0	149	68.4	0.0	19

		**Bovine**			**Swine**	

Ampicillin	15	15	100	62.5	62.5	24
Cefotaxime	10	10	100	0	0	24
Chloramphenicol	10	10	100	20.83	20.83	24
Ciprofloxacin	10	0	100	58.33	20.83	24
Colistin	10	10	100	0	0	24
Gentamicin	15	5	100	20.83	0	24
Meropenem	0	0	100	0	0	24
Streptomycin	20		100	62.5		24
Sulfamethoxazole	95		100	100		24
Tetracycline	25		100	62.5		24
Trimethoprim-sulfamethoxazole	25	0	100	58.33	0	24

### Overall resistance

For most antimicrobials, resistance levels were in the following descending order: Turkey-chicken-cow-pig. High rates of microbiological resistance (more than 30% of resistant isolates) in isolates from chicken and turkey were shown for ampicillin, chloramphenicol, ciprofloxacin, colistin, streptomycin, sulfamethoxazole, trimethoprim-sulfamethoxazole, and tetracycline, and high rates of clinical resistance – for ampicillin, chloramphenicol, ciprofloxacin, and colistin. The highest prevalence of resistance (both clinical and microbiological) was shown for ciprofloxacin – more than 60%. For almost all antimicrobials, the resistance of isolates from turkey was higher than that from chicken, but a small number of isolates from turkey should be taken into account. The lowest resistance (both clinical and microbiological) was shown for cefotaxime and meropenem. No isolates resistant to meropenem were found, which is consistent with the absence of approval for the use of carbapenem in food-producing animals in Russia. Resistance to gentamicin was low in isolates from chicken but high in isolates from turkey.

In isolates from pigs, high levels of microbiological resistance (more than 30%) were found for ­ampicillin, ciprofloxacin, gentamicin, streptomycin, sulfamethoxazole, trimethoprim-sulfamethoxazole, and tetracycline, whereas a high level of clinical resistance was shown only for ampicillin. Zero microbiological resistance was found for cefotaxime and meropenem, zero clinical resistance – for cefotaxime, meropenem, gentamicin, and trimethoprim-sulfamethoxazole.

In isolates from cows, the level of ­resistance was generally low; only for sulfamethoxazole, 95% of isolates were found to be microbiologically resistant. All isolates were clinically susceptible to meropenem, ciprofloxacin, and trimethoprim-sulfamethoxazole.

WHO and OIE state that in terms of *E. coli* resistance, the use of fluoroquinolones, third or higher generation cephalosporins, and colistin in animals poses the highest risk for public health and should be limited to therapeutic use [21,22]. In Russia, these drugs are approved for treatment and prevention. Therefore, we studied the resistance to colistin, cefotaxime (third-generation cephalosporin), and ciprofloxacin (fluoroquinolone) in more detail.

### Resistance to colistin

**Figure-1:**
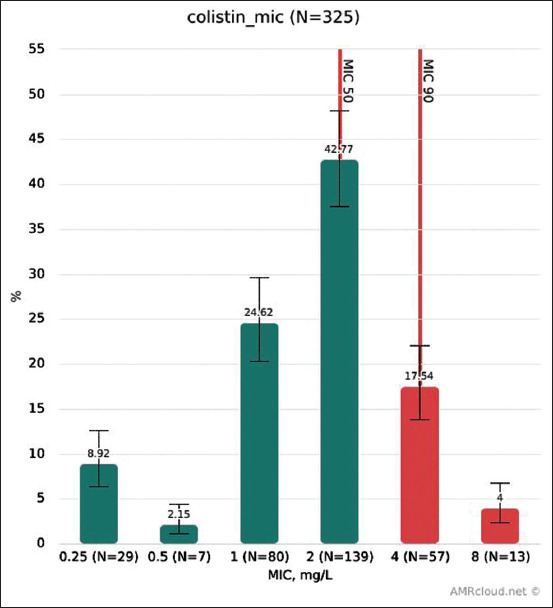
Colistin MIC distribution.

To prove the significance of results for colistin, AST was carried out not only using the manual broth microdilution method (with appropriate quality control) but also using an automated Sensititre™ system (Thermo Fischer Scientific, USA) with colistin-containing plates. The results for isolates tested by both methods were completely consistent; therefore, the data for the two methods were merged. Colistin MIC distribution is shown in Figure-1.

**Figure-2:**
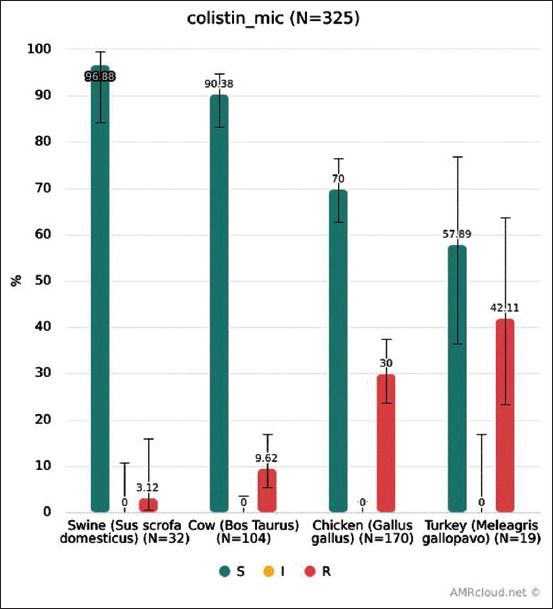
Colistin resistance of *Escherichia coli* from each animal species.

For colistin, the same breakpoint, 2 μg/L, is internationally used to determine both clinical (EUCAST and CLSI) and microbiological resistance. Resistance to colistin of isolates from each animal species is shown in Figure-2. The highest level was found in isolates from poultry, especially from turkey. However, it should be taken into account that from turkey we collected a relatively small number of isolates. The largest number of resistant isolates was found in Belgorod Oblast (43 isolates). However, they were also found in other parts of Russia: In Penza Oblast (10), Kaluga Oblast (5), Chelyabinsk Oblast (5), Novosibirsk Oblast (5), Tambov Oblast (1), and Irkutsk Oblast (1), which indicates that resistance to colistin was not a local trend. Nevertheless, the biggest contribution was made by three intensive breeding farms in Belgorod Oblast.

### Resistance to cefotaxime

**Figure-3:**
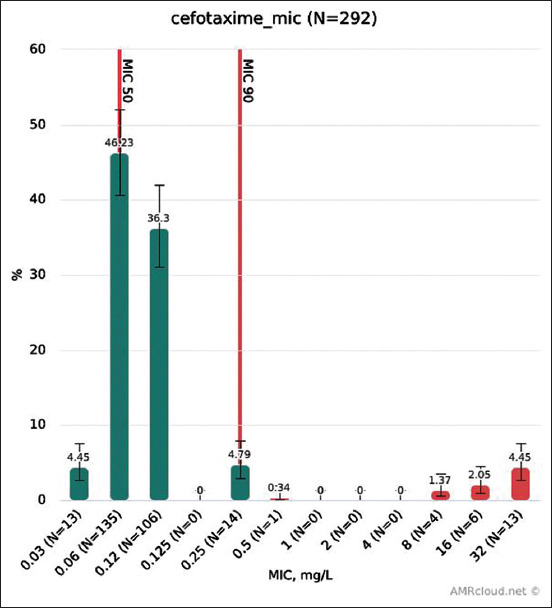
Cefotaxime MIC distribution.

**Figure-4:**
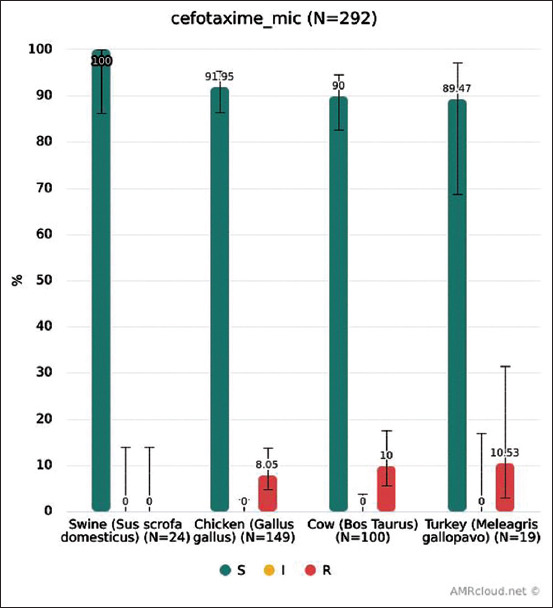
Cefotaxime resistance of *Escherichia coli* from each animal species.

MIC distribution for cefotaxime is shown in Figure-3. Microbiological resistance (EUCAST ECOFFs) to cefotaxime of isolates from each animal species is shown in Figure-4. No cefotaxime-resistant isolates were found in pigs; for other species, the levels of resistance were similar (8-10%). All isolates from poultry resistant to cefotaxime were collected at two intensive breeding farms in Belgorod Oblast, from cows – in two regions, Kaluga and Tver Oblasts.

### Resistance to ciprofloxacin

**Figure-5:**
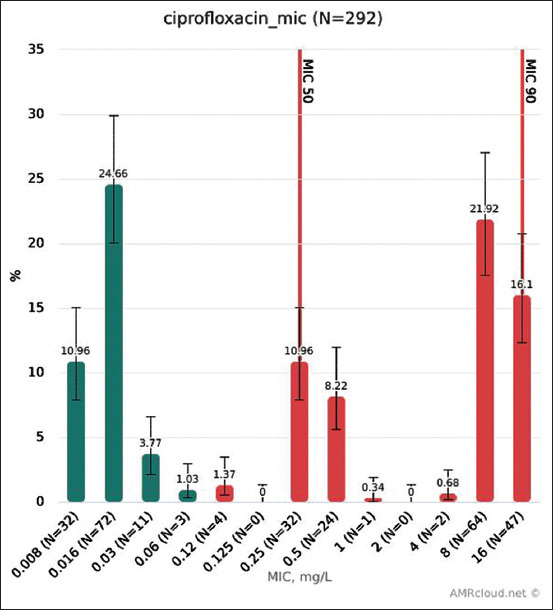
Ciprofloxacin MIC distribution.

**Figure-6:**
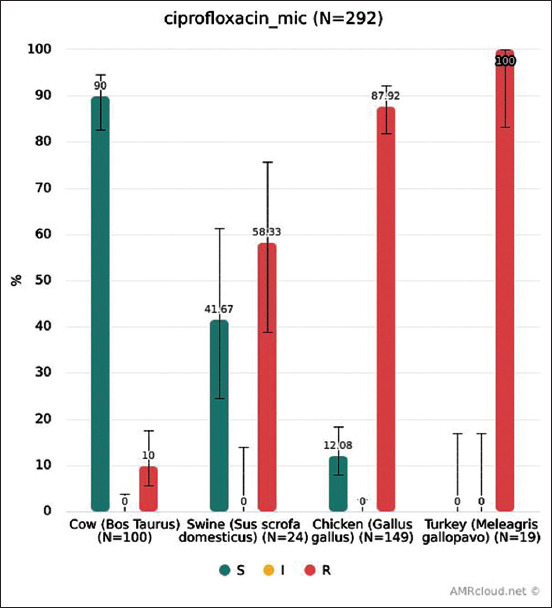
Ciprofloxacin resistance of *Escherichia* *coli* from each animal species. The red bar in the resistance prevalence charts represents the percentage of resistant isolates, the green bar – the percentage of sensitive isolates. “n” means the number of isolates tested. The black thin line in the bars represents the 95% confidence interval.

MIC distribution for ciprofloxacin is shown in Figure-5. Microbiological resistance (EUCAST ECOFFs) for ciprofloxacin of isolates from each animal species is shown in Figure-6. The highest level of resistance was found in isolates from poultry.

### Multidrug resistance

The percentage of isolates with simultaneous resistance to three or more classes of antimicrobials is presented in Table-4. More than 50% of isolates from poultry had microbiological resistance to six classes. All isolates from chicken, turkey and pigs can be classified as “multidrug-resistant” in terms of microbiological resistance, while less than half of isolates showed simultaneous clinical resistance to three classes of antimicrobials. Isolates from cows showed the lowest level of multidrug resistance. No pan-resistant isolates were discovered.

**Table 4:** Multidrug resistance among isolates from different animals.

Microbiological resistance				Clinical resistance	

Broiler chicken					

Simultaneous resistance	% Of resistant isolates	95% Confidence interval range, %	Simultaneous resistance	% Of resistant isolates	95% Confidence interval range, %
3 classes	94.63	89.76-97.25	3 classes	44.59	36.82-52.64
4 classes	79.87	72.71-85.52	4 classes	16.89	11.71-23.75
5 classes	72.48	64.82-79.02	5 classes	4.05	1.87-8.56
6 classes	51.01	43.06-58.91	6 classes	0	0-2.53
7 classes	26.17	19.78-33.77			
8 classes	6.71	3.69-11.91			
9 classes	0.67	0.12-3.7			
10 classes	0	0-2.51			
Turkey					
3 classes	100	83.18-100	3 classes	47.37	27.33-68.29
4 classes	89.47	68.61-97.06	4 classes	36.84	19.15-58.96
5 classes	68.42	46.01-84.64	5 classes	31.58	15.36-53.99
6 classes	57.89	36.28-76.86	6 classes	0	0-16.82
7 classes	36.84	19.15-58.96			
8 classes	21.05	8.51-43.33			
9 classes	0	0-16.82			
Cow					
3 classes	20	13.34-28.88	3 classes	10	5.52-17.44
4 classes	20	13.34-28.88	4 classes	5	2.15-11.18
5 classes	15	9.31-23.28	5 classes	5	2.15-11.18
6 classes	10	5.52-17.44	6 classes	0	0-3.7
7 classes	10	5.52-17.44			
8 classes	5	2.15-11.18			
9 classes	5	2.15-11.18			
10 classes	0	0-3.7			
Swine					
3 classes	100	100	3 classes	0	0-13.8
4 classes	41.67	24.47-61.17			
5 classes	41.67	24.47-61.17			
6 classes	41.67	24.47-61.17			
7 classes	0	0-13.8			

All the data on the prevalence of resistance can be found at the AMRcloud online platform in our open access project: https://amrcloud.net/en/project/vgnki/

## Discussion

### Comparison of *E. coli* resistance to colistin, cefotaxime, and ciprofloxacin in Russia with data from other countries

The prevalence of colistin resistance in *E. coli* isolates from broiler chicken in our study was 30% (95% confidence interval [CI]: 23.62-37.27%). This is significantly higher than 2% of the overall resistance shown in the EU in 2016 [8]. According to the EU reports, the overall resistance was calculated by combining all isolates from all member states, so the differences between countries in the numbers of isolates tested should be taken into account. However, studies in several countries indicated much higher levels of resistance, in particular, in China in 2015: About 25% for *E. coli* from broilers from a surveillance program [11] and 35% from intensive breeding farms in one of Chinese provinces [23]. It should also be noted that in the first study, the authors found a clear trend of increasing colistin resistance of *E. coli* from animals between 2008 and 2015.

According to our data, the colistin resistance of *E. coli* from pigs was 3.12% (95% CI 0.55-15.74%), which is also higher than in the EU in 2017 (0.3% in total) [12]. However, the aforementioned surveillance study from China showed a much higher level of colistin resistance in *E. coli* from pigs, 46% in 2015 [9], while a year earlier, it was 10% lower. A study in one province of China showed 54% colistin resistance of *E. coli* from pigs in 2016 [23].

For cefotaxime, the EUCAST and CLSI clinical breakpoints are also internationally agreed on (MIC>2 μg/ml defines an isolate as resistant); by comparison, the EUCAST ECOFF (microbiological breakpoint) is 0.25 μg/ml.

In our study, 8.05% (95% CI: 4.67-13.55%) of isolates from broiler chicken were microbiologically resistant to cefotaxime. A 2017 EU report shows that the overall level of microbiological resistance in *E. coli* from broilers was 4% with much higher levels for some EU members, for example, 6.2% in Portugal and 52% in Lithuania (the highest level) [8]. In our study, 7.38% (95% CI: 4.17-12.74%) of isolates from chicken showed clinical resistance to cephotaxime, while other studies found that 23% of *E. coli* isolates from broilers had clinical resistance in Brazil, about 25% in Spain, and more than 50% in China [12]. In pigs, we did not find isolates resistant to cefotaxime (95% CI: 0-13.8%), whereas in the EU, 1.4% of microbiologically resistant *E. coli* from fattening pigs was reported in 2017 [9].

In our study, the microbiological resistance of isolates from broiler chicken to ciprofloxacin was 87.92% (95% CI: 81.71-92.22%). The overall resistance of isolates from broilers reported by 27 EU members in 2016 was 64%, which is also relatively high: About 94% in Portugal, 91% in Spain, 90% in Poland, 87% in Bulgaria, etc. [8]. We can also compare the resistance to ciprofloxacin of *E. coli* isolates in Russia to that in the USA. The overall resistance of *E. coli* from poultry + cow + pig in Russia using CLSI breakpoint was 38%, while, according to the NARMS 2015 report, the resistance of *E. coli* from each animal species to ciprofloxacin did not exceed 3% [24]. This may be due to the limited use of fluoroquinolones for animals in the USA. MIC distribution for ciprofloxacin is shown in Figure-5.

### Combined *E. coli* resistance to ciprofloxacin and cefotaxime

Combined resistance of commensal *E. coli* isolates to ciprofloxacin and cefotaxime is included in the EU integrated surveillance programs [8,9] and is an important indicator of resistance, since both drugs are referred to as critically important for the human medicine according to the WHO [21].

In our study, simultaneous clinical resistance to ciprofloxacin and cefotaxime was found in 6.04% of isolates from chicken (95% CI: 3.21-11.08%), in 0% from pigs (95% CI: 0-13.8%), and in 0% from cows (95% CI: 0-3.56%). For reference, in the EU as a whole in 2016-2017, the corresponding values were 1.2% for isolates from broiler chicken (up to 8% for some countries), 0.4% from pigs, and 0.5% from calves [8,9].

Most isolates from poultry, including those resistant to critically important antimicrobials, were collected at three intensive breeding farms in one region; Belgorod Oblast (Figure-2), one of the most agriculturally developed regions of Russia. These farms are likely to be representative of all intensive breeding farms in the country. However, the results for turkey should be treated with caution because all isolates were collected at one farm. Further research is required.

### Comparison of *E. coli* resistance to other antimicrobials with data from other countries

Microbiological resistance to other antimicrobials in Russia was compared with data from the EU [8,9]. In general, the EU reports show a high variability for all antimicrobials among EU countries: There are countries with rather low levels of resistance, for example, the Scandinavian countries, and with high levels, such as Spain, Poland, Lithuania, Italy, and Cyprus. It should be taken into account that the data from countries with generally low levels of resistance and policies of rational use of antimicrobials in animal husbandry make a significant contribution to the overall resistance levels calculated for the EU.

For isolates from broiler chicken, the resistance levels for gentamicin, chloramphenicol, and sulfamethoxazole in Russia were higher than in the EU as a whole and closer to the EU members with the highest level of resistance, while for meropenem, tetracycline, and ampicillin, the resistance levels in Russia were similar or even lower than the overall levels in the EU (47% for ampicillin vs. 58% in the EU).

For cows (adults+calves), our data were compared with the EU data on calves. Again, we found that the resistance levels for ampicillin and tetracycline were much lower than those in the EU as a whole (15% vs. 29% for ampicillin and 25% vs. 44% for tetracycline), whereas for ciprofloxacin, meropenem, and chloramphenicol the resistance levels in Russia were close to the overall EU levels. For gentamicin and sulfamethoxazole, the resistance levels in Russia were higher than in the EU.

## Conclusion

Here, we demonstrate that the resistance of *E. coli* in isolates from poultry to most antimicrobials appears to be higher than that from pigs, which is still higher than that from cattle. This is consistent with the results for *E. coli* in the EU [8,9].

In Russia, we found high levels of resistance to critically important antimicrobials, including colistin, ciprofloxacin, cefotaxime, and ciprofloxacin-cefotaxime combination, primarily in isolates from poultry, chicken, and turkey. Comparable high levels of resistance to critically important antimicrobials were found in other countries, for example, China, Brazil, and Spain.

At the same time, resistance to other antimicrobials such as ampicillin and tetracycline was found to be similar or even lower than in the EU.

The National Plan for Implementation of the Strategy to Minimize and Contain AMR for the Period until 2030 was approved by the Government of Russia in 2019 [13]. One of the key points of the plan is establishing the list of antimicrobials to be restricted for use in animals. Data on the resistance of commensal bacteria collected from farm animals to various antimicrobial agents may be useful for prioritizing the antimicrobials to reduce their use in agriculture.

 According to our data, application of antimicrobials such as colistin, fluoroquinolones, and cephalosporins of last generations (3^rd^-5^th^) may be limited first of all by means of partial substitution for other antimicrobials of the lower importance for medicine and lower levels of resistance, such as penicillin and tetracycline.

To fully address this issue, further research is required on the AMR of bacteria from food-producing animals and food products, including other commensal bacteria, such as enterococci, and pathogenic zoonotic bacteria, such as *Campylobacter* and Salmonella.

## Author’s Contributions

DAM performed data analysis and wrote the manuscript. OEI designed the protocol. SYK performed antimicrobial susceptibility testing, isolation, and sampling. AVP performed isolation and sampling. MAG supervised the work. All authors read and approved the final manuscript.
